# Exposure to Adversity and its Impact on Later Life Cognitive, Mental, and Physical Health

**DOI:** 10.3389/ijph.2024.1606499

**Published:** 2024-06-19

**Authors:** Elyse A. Jennings, Sumaya Mall, Darina T. Bassil, Kathleen Kahn

**Affiliations:** ^1^ Center for Population and Development Studies, Harvard T.H. Chan School of Public Health, Boston, MA, United States; ^2^ Division of Epidemiology and Biostatistics, School of Public Health, University of the Witwatersrand, Johannesburg, South Africa; ^3^ MRC/Wits Rural Public Health and Health Transitions Research Unit (Agincourt), School of Public Health, Faculty of Health Sciences, University of the Witwatersrand, Johannesburg, South Africa

**Keywords:** middle and late adulthood, South Africa, adverse experiences, adverse childhood experiences, cognitive health, mental health, physical health

## Abstract

**Objectives:**

We aimed to assess later-life health responses to childhood and lifetime adversity in a cohort of rural, Black South African adults.

**Methods:**

We performed ordinary least squares regression using two waves of data from Health and Aging in Africa: A Longitudinal Study of an INDEPTH Community in South Africa (HAALSI) to estimate a decline in cognitive, mental, and physical health over approximately 3 years. Our analytic sample consisted of 1,993 women and 1,496 men.

**Results:**

Associations between several types of adversity and health outcomes point to declines in health. At the same time, many adverse experiences are associated with improvements in cognitive, mental, and physical health in later life. The direction of the association varied by type of exposure, health outcome, and gender.

**Conclusion:**

In populations exposed to many adversities during life, specific adverse experiences may sometimes be associated with greater improvements (and not just greater decline) in health in later life. Further research is needed to unpack the mechanisms at play in these populations.

## Introduction

The events and experiences that individuals encounter throughout their lives can have a profound impact on their health and wellbeing [1–5]. Evidence suggests that adverse experiences in childhood are linked to several health outcomes in adulthood, such as cardiovascular disease and premature mortality [6, 7]. Adverse experiences into adulthood also have the potential to affect health in later life [8, 9]. Existing research typically focuses on Western and high income populations, thereby limiting our understanding of the effects of adverse experiences in different cultural contexts and among populations with relatively limited resources.

In this study, we focused on a population of rural Black South Africans and examine the association between adversity—both during childhood and across the life course—and the rate of decline in cognitive, mental, and physical health in later adulthood. We investigate the impact of specific domains of adverse experiences and exposures: adverse experiences in the childhood home, the experience of having a spouse or child with a drug or alcohol addiction, exposure to violence (both in combat and non-combat), the experience of assault, and exposure to natural disasters. Importantly, this research sheds light on these associations in a population that was affected by structural racism under apartheid for much of its life, and still experiences significant poverty. These experiences, in themselves, have been linked to poor health outcomes in multiple settings, including the United States [10–12]. In the population we focus on there is a high burden of morbidity and mortality [13, 14]. Uncovering associations between specific adverse exposures and health outcomes will offer insight into the extent to which adversity may impact this burden.

Extensive research has documented the significance of adverse experiences and exposures across the lifecourse for health outcomes [5, 9, 15, 16]. Adversity experienced during childhood can negatively affect mental health, cardiovascular disease, morbidity, and the risk of mortality in adulthood [5–7, 15–17]. For example, there is evidence that mental health disorders play an important mediating role in the association between various adverse childhood experiences and cardiovascular outcomes [18], and that greater exposure to childhood adversity may increase the risk of heart failure [17]. Adversity has also been linked to greater depressive symptoms [19, 20]. Other research has suggested that children from homes with more parental turmoil are at greater risk of poor academic and behavioral outcomes [21], which in turn may affect cognitive and mental health. However, existing studies often find null associations between childhood adversity and cognition in later life [5, 22–24]. Mechanisms through which adversity in childhood may impact health in later life include prolonged stress on the body, brain development, and other biological mechanisms that have the potential to affect social and emotional development, as well [7]. Therefore, we might expect to see worse mental and physical health outcomes in later life in individuals exposed to adversity in their childhood, and either worse or similar cognitive health when compared with those who did not experience the same adversity.

As people enter adulthood, their experiences and exposures to new adverse events can also have long-term impacts. Exposure to adversity across the life course has the potential to profoundly impact cognitive, mental, and physical health [8, 9, 25–29]. For example, having a close relative who is addicted to drugs or alcohol has been found to have lasting effects on an individual’s emotional well-being, often through stress and family strain [30, 31]. Witnessing violence or being physically or sexually assaulted can result in prolonged stress, and has been linked to poor mental and physical health outcomes [32–38]. Natural disasters—adversity that is experienced by a community—can also affect the mental and physical well-being of individuals by disrupting social processes and limiting access to medication or health care [9, 25, 29, 39]. The evidence for the impacts of adversity on cognitive outcomes is more mixed [40–42], and these impacts may vary depending on the type of adversity experienced, the timing of the exposure, and the cognitive domains under investigation [41, 43]. Proposed mechanistic pathways linking lifetime adversity to cognition include stress-induced effects on brain structures and depression [20, 44].

Responses to trauma may differ according to gender [9, 45–47]. For example, studies have found that women may be less resilient than men after exposure to a natural disasters [9, 48], and that women may suffer more and for longer than men after adverse experiences [45]. Evidence suggests that women tend to have internalized responses to adverse exposures, whereas men exhibit externalized responses more often [46], which may have the potential to differentially affect which aspects of health are impacted in later life. Women and men may also differ in their access to support that can help protect against the negative consequences of adversity—women have often been found to have larger social networks than men [49, 50], although evidence from South Africa suggests that men have relatively large social networks [51].

We expect that each adverse experience/exposure will be associated with an accelerated decline in cognitive, mental, and physical health for both men and women in this population. Given past findings, we expect the strongest associations for mental health decline, and weaker associations for cognitive decline [5, 9, 18, 22–24, 34]. As responses to trauma may be inextricably linked to gender, we empirically tested these expectations separately for women and men.

Our investigation revealed mixed findings, with certain adverse experiences and exposures associated with improvements in health, while others were found to be associated with the expected decline in health. These mixed results are apparent for both women and men. The unique lifetime experiences of this population may have led to greater resilience and the effective development of coping mechanisms in the face of the adversities we analyzed. At the same time, many adverse events are associated with a decline in cognitive, mental, and/or physical health, pointing to the importance of understanding mechanisms for intervention.

## Methods

The study area for this investigation was a cluster of villages in and near Agincourt, South Africa: a rural, low-income area in the Bushbuckridge sub-district of Mpumalanga Province in north-eastern South Africa. Agincourt is located in an area of forced racial segregation during apartheid, where Black South Africans belonging to the Shangaan ethnic group were forcibly moved [52, 53]. Mpumalanga is the second most homogeneous province in South Africa, with over 90% of residents identifying as Black African [54]. The Agincourt area is also home to many former Mozambican refugees, the older of whom are likely to have been exposed to combat violence during the years of civil war in Mozambique (1976–1992). While South Africa is one of the more economically advanced countries in the region, much of the population of Agincourt is poor [55].

### Data

We used data from Health and Aging in Africa: A Longitudinal Study of an International Network for the Demographic Evaluation of Populations and Their Health (INDEPTH) Community in South Africa (HAALSI). The HAALSI study is a population-based survey that aims to characterize a cohort of men and women aged 40 years and older in rural South Africa with respect to health and aging [55]. Participants were sampled from the Agincourt Health and socio-Demographic Surveillance System (Agincourt HDSS) in Mpumalanga province [56]. The identified sampling frame consisted of 8,974 women and 3,901 men who were aged 40 years or older as of 1 July 2014 and who had resided continuously in the study area for the 12 months prior to the 2013 Agincourt census. Using gender-specific sampling fractions to ensure a gender-balanced cohort, 6,281 participants were randomly selected to participate in HAALSI.

Baseline (wave 1) interviews were completed between November 2014 and November 2015, with a total of 5,059 respondents (response rate of 85.9% among eligible individuals). Wave 2 interviews were conducted between October 2018 and November 2019 with all living members of the 5,059 individual cohort (response rate of 94%; *n* = 4,176). Wave 3 interviews were conducted between July 2021 and March 2022 among living members of the original cohort (response rate of 93%; *n* = 3,707). In wave 2, all respondents were administered a life history module that included questions about childhood and lifetime adversity. Our investigation focused on responses to these life history items to predict the decline in health between waves 2 and 3. Estimates of change over this short period of approximately three years are likely conservative.

### Measures

#### Dependent Variables

To assess the decline in cognitive health between waves 2 and 3, we developed a composite measure to indicate a decline in cognitive function. In each wave, we summed the total immediate recall of 10 words over three trials (up to 10 points per trial), delayed recall of these 10 words (up to 10 points), and orientation (correctly stating the year, month, day, and name of the current South African president; up to 4 points) [57]. The total score in each wave totaled a maximum of 44 points. We then coded the measure of cognitive decline by subtracting the composite score in wave 3 from the wave 2 score.

Next, we coded a measure to indicate a decline in mental health between waves 2 and 3 using the 20-item Center for Epidemiologic Studies Depression (CES-D) scale [58]. The scale included items asking how often the respondents had experienced different depressive symptoms in the previous week (e.g., felt bothered, had poor appetite, had trouble concentrating, etc.). Response options included “rarely or none of the time (less than 1 day),” “some or little of the time (1-2 days),” “occasionally or a moderate amount of time (3-4 days),” and “most or all of the time (5–7 days).” Following other studies, these options were coded from 0 to 3 and then summed to produce a score of 0-60 at each wave [59]. We then coded a measure to indicate a decline in mental health (i.e., an increase in depressive symptoms) by subtracting the CES-D score at wave 2 from the score at wave 3.

Finally, we estimated the decline in physical health between waves 2 and 3. For each wave, we coded a measure of the sum of activities of daily living (ADLs) that the respondents reported having difficulty with (i.e., walking, bathing, eating, getting out of bed, and using the toilet). To measure the decline in physical health (i.e., an increase in physical limitations), we subtracted the sum of reported difficulties in up to five ADLs at wave 2 from the sum of the same at wave 3.

#### Independent Variables

We coded a series of measures to indicate lifetime exposure to adverse events or experiences from the wave 2 survey. We grouped items where appropriate, according to the results of factor analyses. First, a measure of exposure to adverse family experiences in childhood was coded as 1 if the respondents reported experiencing at least one of the following before the age of 16: parents arguing or fighting often; parents drinking excessively, taking drugs, or having mental health problems; or the respondents being physically abused by their parents. If the respondents reported none of these experiences, the value was set to 0.

A measure indicating whether the respondents ever had a spouse, partner, or child who was addicted to drugs or alcohol was coded 1 if yes and 0 if no.

Next, we coded two measures to indicate exposure to violence. The first measure indicates exposure to violence other than in combat, and was coded 1 if the respondents reported ever witnessing an accident or violent act in which someone was killed or seriously wounded other than in combat or in the military, and 0 if not. The second was a measure indicating exposure to violence in military or combat, coded as 1 if the respondents ever fired a weapon in combat, were shot at, or witnessed someone being seriously injured or killed in war or military action, and coded 0 if not.

A measure of experience of assault indicates the respondents’ direct experience of ever having been the victim of a serious physical attack or assault, or ever having been the victim of sexual assault. If the respondents experienced either type of assault they received a code of 1; if not, the value was set to 0.

Finally, a measure indicating the respondents’ exposure to natural disasters was coded as 1 if they reported having ever experienced a major flood, fire, earthquake, or other natural disasters, and 0 if they reported that they did not.

#### Covariates

We also controlled for a series of variables that may impact the relationship between childhood exposures and adult health. First, we controlled for family background with binary measures of whether the respondents reported that their parents were in a union when they were born and whether their father attended school. We also controlled for a measure indicating whether the respondents were born outside South Africa. Next, we controlled for the respondents’ reports of their childhood health, coded as 1 if they reported poor, fair, or good health, 2 if they reported very good health, and 3 if they reported excellent health in childhood.

Next, we controlled for the respondents’ (continuous) age at wave 1. We also controlled for a series of three dummy variables to indicate their educational attainment, coded 1 if they had not completed any formal education, 2 if they had some or completed primary education, or 3 if they had some secondary education or higher at wave 1. We also controlled for respondents’ employment status at wave 2, coded as four dummy variables to indicate: (1) employed, (2) homemaker (translated to be understood as “home manager”), if they did not report being employed, (3) retired, if they did not report being employed or a home manager, or (4) not working, if they did not report that they were any of the above statuses.

We then controlled for the respondents' marital status at the time of the wave 2 survey, with a series of dummy variables indicating whether the respondents were (1) never married, (2) married or living with a partner, (3) separated or divorced, or (4) widowed. Additionally, we controlled for a measure indicating whether the respondents reported having been married more than once at wave 2. We also controlled for the number of children the respondents had at wave 1, which was top-coded at 8 children.

Next, we controlled for household wealth and the frequency of social support, both at wave 2. Households were ranked according to principal component analysis scores for household ownership of items such as televisions, refrigerators, livestock, and vehicles in addition to housing characteristics, type of water, and sanitation facilities [60]. The ranking was then coded into quintiles to indicate the household wealth index. The frequency of social support was coded as the average approximate number of days per month that the respondents reported receiving emotional, physical, and/or informational support from any of up to seven named social contacts.

Finally, in models predicting a decline in cognitive and physical health, we control for depressive symptoms at wave 2 using the 20-item CES-D scale, as described above. We also control for self-reported diagnosis (ever) with stroke in models predicting a decline in physical health, coded as 1 if the respondents reported having ever had a stroke at wave 2, and 0 if not.

### Analysis

We used ordinary least squares (OLS) regression to estimate declines in cognitive, mental, and physical health. We standardized the dependent variables (health outcomes) to allow for greater comparability across models. We used multiple imputations for missing values on any of the independent and covariate measures in our analyses to retain the full sample of respondents who completed the waves 2 and 3 surveys and have no missing information on the outcome measures. This resulted in a base analytic sample of 1,981 women and 1,486 men, which was restricted in each model based on missing data on the respective outcome measure (see Table 1). We applied inverse probability weights to our models to account for mortality between waves 1 and 3.

**TABLE 1:** Analytic sample characteristics from Health and Aging in Africa: A Longitudinal Study of an International Network for the Demographic Evaluation of Populations and Their Health (INDEPTH) Community in South Africa (HAALSI) (Agincourt, South Africa. 2014–2022).

	Women	Men	Difference between women and men
	Mean/proportion (SD)	Mean/proportion (SD)	
*Dependent variables (change between waves 2 and 3, standardized)*			
Decline in cognitive health	−0.03 (0.99)	0.04 (1.01)	0.07 ***
Decline in mental health	−0.03 (0.83)	0.04 (1.20)	0.07 ***
Decline in physical health	−0.02 (0.93)	0.03 (1.09)	0.05***
*Independent variables (wave 2)*			
Adverse family experiences in childhood	0.24	0.22	−0.02 ***
Ever had a spouse, partner or child who was an addict	0.10	0.04	−0.06 ***
Exposure to violence (non-combat)	0.14	0.23	0.09 ***
Exposure to violence (combat)	0.09	0.16	0.07 ***
Experience of assault	0.06	0.09	0.03 ***
Exposure to natural disaster	0.42	0.43	0.01 *
*Covariates*			
Parents not in union at birth	0.07	0.07	0.001
Father attended school	0.27	0.24	−0.03 ***
Born outside of South Africa	0.32	0.29	−0.03 ***
Childhood health	1.89 (0.80)	1.94 (0.80)	0.05 ***
Age (wave 1)	60.43 (12.21)	60.32 (11.70)	−0.11
Education (wave 1)			
No formal education	0.48	0.38	−0.10 ***
Some/completed primary school	0.34	0.37	0.03 ***
Some/completed secondary or higher education	0.18	0.25	0.07 ***
Employment status (wave 2)			
Not working	0.83	0.70	−0.13 ***
Employed	0.14	0.21	0.07 ***
Retired	0.03	0.09	0.06 ***
Marital status (wave 2)			
Married or living with a partner	0.33	0.63	0.30 ***
Never married/single	0.05	0.11	0.06 ***
Separated or divorced	0.12	0.13	0.01 +
Widowed	0.50	0.13	−0.37 ***
Married more than once (wave 2)	0.12	0.39	0.27 ***
Number of children (wave 1)	4.53 (2.05)	4.73 (2.26)	0.20 ***
Household wealth index (wave 2)	3.03 (1.44)	3.01 (1.41)	−0.02
Frequency of social support (wave 2)	11.56 (7.59)	11.30 (7.44)	−0.26 **
CES-D (wave 2)	15.06 (9.44)	13.72 (9.39)	−1.34 ***
Ever had a stroke (wave 2)	0.02	0.02	0.001

N = 1,751 women and 1,263 men for decline in cognitive function; N = 1,780 women and 1,282 men for decline in mental health; and N = 1,947 women and 1,452 men for decline in physical health. N = 1,981 women and 1,486 for all independent variables and covariates.

CES-D, Center for Epidemiologic Studies Depression Scale.

## Results

Table 1 displays the means, or proportions, for all variables in our models in addition to their standard deviation (for non-binary variables). The last column indicates the gender difference and whether these differences are significant. These descriptive statistics indicate that women experienced significantly less decline in their cognitive, mental, and physical health between waves than men. There were also significant gender differences across exposures. Nearly a quarter of women (24%) and 22% of men faced adverse family experiences in childhood. Ten percent of women and only 4% of men reported ever having a spouse, partner, or child who was an addict. Fourteen percent of women and 23% of men reported exposure to non-combat violence, while 9% of women and 16% of men reported exposure to violence in combat. Six percent of women and 9% of men reported experiencing assault. Finally, 42% of women and 43% of men reported exposure to natural disasters. Notably, 32% of women and 29% of men reported being born outside of South Africa; the majority of these individuals are likely to be Mozambican immigrants who entered the country as refugees.

Table 2 displays the results of OLS regressions estimating the decline in each health outcome among women. Results from Model 1 suggest that childhood adverse family experiences, ever having a spouse, partner or child who was an addict, ever having been exposed to combat violence, and ever having been exposed to natural disasters were each associated with greater improvement in cognitive health between waves (β = −0.20 at *p* < .001; β = −0.06 at *p* < .05; β = −0.11 at *p* < .001; and β = −0.07 at *p* < .001, respectively). On the other hand, exposure to non-combat violence was associated with a decline in cognitive function (β = 0.16 at *p* < .001). The experience of assault was not significantly associated with a decline in cognitive health among women.

**TABLE 2:** Ordinary least squares regression predicting decline in cognitive, mental, and physical health among women (Agincourt, South Africa. 2014–2022).

	Model 1 decline in cognitive health	Model 2 decline in mental health	Model 3 decline in physical health
Intercept	−0.51 *** (0.06)	−0.06 (0.05)	−0.89 *** (0.06)
*Independent variables*			
Adverse family experiences in childhood	−0.20 *** (0.02)	0.004 (0.02)	−0.02 (0.02)
Ever had a spouse, partner or child who was an addict	−0.06 * (0.03)	−0.10 *** (0.02)	0.03 (0.03)
Exposure to violence (non-combat)	0.16 *** (0.02)	−0.07 *** (0.02)	−0.10 *** (0.02)
Exposure to violence (combat)	−0.11 *** (0.03)	0.12 *** (0.02)	0.10 *** (0.03)
Experience of assault	−0.05 (0.03)	0.16 *** (0.03)	0.04 (0.03)
Exposure to natural disaster	−0.07 *** (0.02)	−0.001 (0.01)	−0.03 + (0.02)
*Covariates*			
Parents not in union at birth	0.11 *** (0.03)	0.09 *** (0.02)	−0.09 ** (0.03)
Father attended school	0.04 * (0.02)	−0.01 (0.01)	0.03 (0.02)
Born outside of South Africa	−0.001 (0.02)	−0.04 * (0.02)	0.16 *** (0.02)
Childhood health	0.05 *** (0.01)	−0.03 *** (0.01)	−0.01 (0.01)
Age	0.004 *** (0.001)	0.002 *** (0.001)	0.02 *** (0.001)
Education (Ref: No formal education)			
Some/completed primary school	0.22 *** (0.02)	0.10 *** (0.02)	−0.06 ** (0.02)
Some/completed secondary or higher education	0.35 *** (0.03)	−0.05 * (0.02)	0.11 *** (0.03)
Employment status (Ref: Not working)			
Employed	−0.10 *** (0.02)	0.20 *** (0.02)	0.06 * (0.02)
Retired	−0.64 *** (0.05)	−0.04 (0.04)	−0.17 *** (0.05)
Marital status (Ref: Married or living with a partner)			
Never married/single	0.10 ** (0.04)	0.07 * (0.03)	0.04 (0.04)
Separated or divorced	0.02 (0.03)	−0.09 *** (0.02)	−0.08 ** (0.03)
Widowed	−0.01 (0.02)	−0.05 ** (0.01)	−0.03 + (0.02)
Married more than once	−0.14 *** (0.02)	0.02 (0.02)	0.16 *** (0.02)
Number of children	−0.003 (0.004)	−0.01 * (0.003)	−0.02 *** (0.004)
Household wealth index	0.02 *** (0.01)	−0.02 *** (0.01)	0.003 (0.01)
Frequency of social support	−0.002 + (0.001)	0.001 + (0.001)	0.004 *** (0.001)
CESD at wave 2	0.003 *** (0.001)		−0.01 *** (0.001)
Ever had a stroke			−0.02 (0.05)
*N*	*1, 751*	*1,780*	*1,947*

Models weighted for mortality. Beta coefficients with standard errors in parentheses.

Two-tailed tests, + *p*< .10, *p < .05, **p < .01, ***p < .001.

Model 2 indicates that ever having a spouse, partner, or child who was an addict was associated with an improvement in women’s mental health between waves (β = −0.10 at *p* < .001), as was exposure to non-combat violence (β = −0.07, at *p* < .001). Exposure to violence in combat and experience of assault were each associated with a decline in mental health (β = 0.12 at *p* < .001 and β = 0.16 at *p* < .001, respectively). Adverse family experiences in childhood and exposure to natural disasters were not significantly associated with decline in mental health among women.

In Model 3 of Table 2, we found that exposure to non-combat violence and exposure to natural disasters were each associated with improved physical health between waves (−0.10 at *p* < .001, and −0.03 at *p* < .10, respectively) among women. Exposure to violence in combat was associated with a decline in physical health (0.10 at *p* < .001). Adverse family experiences in childhood, ever having had a spouse, partner or child who was an addict, and experience of assault were not significantly associated with decline in physical health among women.

Table 3 estimates these associations among men. Results from Model 1 suggest that adverse family experiences in childhood and exposure to a natural disaster were each associated with an improvement in men’s cognitive health between waves (−0.11 at *p* < .001 and −0.06 at *p* < .01, respectively). Exposure to violence, both combat and non-combat, was associated with a decline in men’s cognitive health (0.15 at *p* < .001 and 0.17 at *p* < .001, respectively). Ever having a spouse, partner, or child who was an addict and ever experiencing assault were not significantly associated with men’s cognitive health.

**TABLE 3:** Ordinary least squares regression predicting decline in cognitive, mental, and physical health among men (Agincourt, South Africa. 2014–2022).

	Model 1 decline in cognitive health	Model 2 decline in mental health	Model 3 decline in physical health
Intercept	−0.43 *** (0.08)	0.19 * (0.08)	−0.10 (0.08)
*Independent variables*			
Adverse family experiences in childhood	−0.11 *** (0.02)	0.01 (0.03)	−0.02 (0.03)
Ever had a spouse, partner or child who was an addict	−0.02 (0.05)	−0.14 * (0.06)	−0.01 (0.05)
Exposure to violence (not combat)	0.15 *** (0.02)	0.06 * (0.03)	−0.10 *** (0.03)
Exposure to violence (combat)	0.17 *** (0.03)	−0.14 *** (0.03)	0.08 * (0.03)
Experience of assault	−0.02 (0.03)	0.29 *** (0.04)	0.01 (0.04)
Exposure to natural disaster	−0.06 ** (0.02)	−0.12 *** (0.02)	−0.03 (0.02)
*Covariates*			
Parents not in union at birth	0.14 *** (0.04)	−0.03 (0.04)	−0.13 *** (0.04)
Father attended school	−0.07 ** (0.02)	−0.07 ** (0.03)	0.10 *** (0.03)
Born outside of South Africa	−0.06 * (0.02)	0.004 (0.03)	0.003 (0.02)
Childhood health	0.11 *** (0.01)	0.002 (0.01)	−0.01 (0.01)
Age	0.001 (0.001)	−0.01 *** (0.001)	0.004 *** (0.001)
Education (Ref: No formal education)			
Some/completed primary school	0.18 *** (0.02)	−0.06 * (0.03)	−0.10 *** (0.02)
Some/completed secondary or higher education	0.28 *** (0.03)	−0.01 (0.03)	−0.11 *** (0.03)
Employment status (Ref: Not working)			
Employed	−0.07 ** (0.03)	0.09 ** (0.03)	0.02 (0.03)
Retired	−0.44 *** (0.03)	0.26 *** (0.04)	0.17 *** (0.04)
Marital status (Ref: Married or living with a partner)			
Never married/single	0.09 * (0.03)	−0.13 *** (0.04)	0.13 *** (0.03)
Separated or divorced	−0.06 * (0.03)	0.17 *** (0.03)	0.21 *** (0.03)
Widowed	−0.03 (0.03)	0.05 (0.03)	0.12 *** (0.03)
Married more than once	−0.04 + (0.02)	0.08 *** (0.02)	−0.12 *** (0.02)
Number of children	0.01 * (0.004)	−0.01 + (0.01)	0.02 *** (0.01)
Household wealth index	0.02 ** (0.01)	0.02 ** (0.01)	−0.03 *** (0.01)
Frequency of social support	0.002 + (0.001)	0.01 *** (0.001)	0.002 + (0.001)
CESD at wave 2	0.002 + (0.001)		−0.01 *** (0.001)
Ever had a stroke			−0.57 *** (0.06)
*N*	*1,263*	*1,282*	*1,452*

Models weighted for mortality. Beta coefficients with standard errors in parentheses.

Two-tailed tests, + *p*< .10, *p < .05, **p < .01, ***p < .001.

Model 2 of Table 3 shows that ever having a spouse, partner, or child who was an addict, exposure to violence in combat, and exposure to a natural disasters were associated with greater improvements in men’s mental health (−0.14 at *p* < .05, −0.14 at *p* < .001, and −0.12 at *p* < .001, respectively). Exposure to non-combat violence and experience of assault, on the other hand, were associated with a decline in mental health between waves (0.06 at *p* < .05 and 0.29 at *p* < .001, respectively). Adverse family experiences in childhood were not significantly associated with a decline in mental health among men.

In Model 3 of Table 3, we found that exposure to non-combat violence was associated with improved physical health among men (−0.10 at *p* < .0001). Exposure to violence in combat, on the other hand, was associated with a decline in men’s physical health (0.08 at *p* < .05). The other experiences and exposures in the model were not significantly associated with physical health among men.

In order to directly assess gender differences in the association of adverse exposures and experiences with health outcomes, we tested these same models with gender interactions (see Supplemental Table S1). These results indicated that exposure to violence in combat had significantly different associations with each of the three health outcomes by gender; adverse family experiences in childhood had significantly different associations with cognitive health by gender; and exposure to non-combat violence, experience of assault, and exposure to natural disasters had significantly different associations with mental health by gender.

We performed a sensitivity analysis to assess whether survivor bias might subtly impact our results. We tested the associations for 40–49-year-olds (the least selective for survivor bias) and for those aged 75+ (the most selective) to assess the severity of this bias, and we found mixed evidence. In some cases, adversity was associated with null results or improvements in health for the older group more than the younger group (which would indicate survivor bias), and in other cases adversity was associated with greater declines in health for this group than for the younger group (see Supplemental Tables S2–S5). It is important to keep in mind that healthier people are more likely to have survived long enough to be selected into our analytic sample, but this selection does not appear to impact our results in a consistent direction.

## Discussion

This investigation focused on understanding how adverse experiences and exposures in childhood and throughout life might be associated with the rate of decline in cognitive, mental, and physical health over a period of approximately 3 years in a cohort of middle-aged and older rural Black South Africans. We expected that each adverse experience and exposure would be associated with a decline in health outcomes, although the association may be weaker than in high-income populations. While we did find that some adverse exposures were associated with declines in certain health outcomes, we also found that some of the adverse exposures that we investigated were associated with an improvement in certain measures of health. Furthermore, these associations sometimes differed by gender. These findings are mixed with respect to our expectations and comparability with past research.

Our findings on how exposure to violence in combat and the experience of assault are associated with health outcomes complement existing research. For example, Axinn et al (2023) found, that exposure to civil violence in 7 countries that experienced civil violence since WWII, was associated with a higher risk of mental disorders in the long term [28]. Similarly, in a study of U.S.-based WWII and Korean War veterans, Elder and Chipp (1989) found that heavy combat veterans were at higher risk for emotional and behavioral problems, compared with noncombat and light combat veterans [8]. Interestingly, this study found that those exposed to heavier combat exhibited greater resilience. Our results also suggest a decline in health in response to exposure to combat violence: This exposure was associated with a decline in women’s mental and physical health, and in men’s cognitive and physical health. Other health outcomes, on the other hand, point toward improvements in health as a response to this exposure: women exposed to combat violence experienced improved cognitive health and men exposed to this type of violence experienced improved mental health.

Previous studies have found that intimate partner violence (IPV) leads to worse mental health outcomes, especially for women [34–36]. Similarly, in our study, we found that the experience of assault was associated with worse mental health outcomes for both men and women. Therefore, the experience of assault may have a similar negative impact on mental health across settings. On the other hand, in contrast to this literature, our supplementary analyses found that the decline in men’s mental health is significantly greater than the decline in women’s mental health in response to having experienced assault. This may, in part, be due to measurement: our measure of experience of assault may include not only IPV but also serious physical attacks, which could impact men more severely than women.

Our findings for the other exposures we investigated did not align as well with existing research. For example, childhood adversity and trauma have been linked to a higher risk of mortality [7], mood disorders [2], and cardiovascular and heart disease [6, 17, 18]. On the other hand, studies—including those of a subset of the adults in our sample—have found that childhood adversity has null associations with cognitive health [5, 22–24]. In contrast to each of these previous studies, our analyses revealed significant associations with (improved) cognitive health, but not with mental or physical health. The significant results in our investigation may reflect our focus on changes in health over a three-year period, rather than a cross-sectional measure of cognitive health or a measure of change over a longer period.

Past studies—involving populations in Singapore, Mexico, England, Australia, and Italy—have found that having family members with addiction can lead to poor health outcomes, operating through mechanisms such as stress and family strain [30, 31]. Our investigation revealed the opposite: women who have experienced having a family member with addiction were observed to have improved cognitive health, and both men and women who endured this experience were observed to have improved mental health.

Research on the health impacts of exposure to non-combat violence has tended to focus on mental health, and findings have largely indicated a negative association [32, 33]. Our investigation found more mixed results: men exposed to non-combat violence were found to experience a decline in mental health, but their female counterparts were found to experience improved mental health. Additionally, we found that this exposure may lead to improved physical health, but decreased cognitive health for both men and women.

Existing studies of the health impacts of exposure to natural disasters have also tended to focus on mental health, finding that this exposure is a risk factor for poor mental health [9, 61]. In our investigation, we found null results for the association between this exposure and women’s mental health, but evidence that exposure to natural disasters may lead to improved mental health for men. We also found evidence of improved cognitive health for women and men exposed to natural disasters, and improved physical health for women. These findings are contrary to expectations based on existing research and suggest that resilience may be strong in the HAALSI cohort.

The improvements in health that we found in response to adversity are likely an artifact of the unique disadvantages faced by this population. Exposure to adversity can foster the development of resilience mechanisms and result in improved coping mechanisms [62], which can lead to improved health, particularly if observed over a specific period of time. The majority of the adults in our sample experienced apartheid, while the others experienced the Mozambican civil war before entering South Africa as refugees. This background of adversity, compounded with the specific experiences and exposures we have assessed in this investigation, may have led to hyper-resilience.

Exposure to adversity may enhance coping mechanisms that operate through psychological, biological, behavioral, and/or social processes [9, 62]. For example, people exposed to adversity may cope by ensuring that they have supportive social networks [63, 64], and it is possible that social networks are more responsive in this setting than in others [31]. People may also adopt healthier behaviors as a coping mechanism [62], which could result in improved physical health. Adversity may also trigger changes in stress hormone levels, and alter neural pathways that are related to resilience [65–67], which could have a positive impact on cognitive health. Each of these possible mechanisms, in conjunction with individual differences, genetic predispositions, and socioeconomic factors, may mitigate the negative impact of adversity and promote resilience. Further research is needed to identify and disentangle whether these and/or other mechanisms are at play.

Our results suggest that gender may play an important role in how individuals respond to different types of adversity. The gender differences we found were not always in the same direction, which highlights the complicated gendered processes that underlie these associations. In some cases, these gender differences may be the result of different circumstances and opportunities for exposure [68]. For example, in the case of responses to exposure to combat violence—which we found to be associated with significantly worse cognitive and physical health outcomes for men than for women, but worse mental health outcomes for women than for men (see Supplemental Table S1)—it is important to keep in mind that women are less likely than men to be engaged in combat and more likely to witness it. These unique circumstances may result in different cognitive, mental, and physical processes for women than for men.

Even when women and men have similar exposures, associations with health outcomes may differ due to different biological and/or social mechanisms. For example, women may respond more severely to traumatic experiences [69], and men may respond more through externalized symptoms [46]. These gendered processes may help to explain why we see better health outcomes for men in some cases, such as in our finding that women’s mental health is more negatively impacted by exposure to natural disaster than men’s mental health—a finding that is aligned with results from previous work [9, 48]. On the other hand, women may be better positioned to rely on social support than men, and their social networks may be larger [64, 70]. These different social mechanisms may help explain why we see relatively better outcomes for women in some cases, such as in their mental health responses to non-combat violence and experiences of assault. In fact, some evidence suggests that women may exhibit resilience and post-traumatic growth in response to adversity [71, 72]. Future work would benefit from examining how the mechanisms connecting adversity to health may differ by gender in this setting.

Our expectation that health decline would be most apparent in mental health [5, 9, 23], and least apparent in cognitive health [5, 9, 18, 22–24, 34] was not supported. Mental health was significantly associated with a few of the experiences and exposures that we investigated, but in many cases the association pointed toward improved mental health. Cognitive health was significantly associated with many of the experiences and exposures we investigated, but often in the direction of improved cognitive health. These findings, often in contrast to existing research, further reflect the unique experiences and possible resilience of rural Black South African populations.

This study had limitations that need to be considered. One important limitation is the generalizability of these results. In situating our results within the broader literature, it is apparent that this population is sometimes unique in its health responses to adversity. HAALSI is a population-based sample from the Agincourt Health and socio-Demographic Surveillance System (HDSS) in Mpumalanga province [56], which is nested within INDEPTH [73] and the South African Population Research Infrastructure Network (SAPRIN) [74]–larger research networks in sub-Saharan Africa, that allow for comparisons and extrapolation both within and beyond South Africa. Analytical work has demonstrated similarities in demographic structure and health status across rural commuties in sub-Saharan Africa [74], indicating that our findings are likely to be generalizable to other rural, resource-limited areas in the region.

Concerns about survivor bias are a second important limitation that applies to all studies, and especially to studies of aging like this one. The sensitivity analyses that we performed did not eliminate this concern. Those who have survived long enough to be selected for the sample may have characteristics that protect them against rapid health decline, which would make our estimates of decline more conservative. Third, we focused on changes in health over approximately three years, which limited the amount of change we were able to observe. Nonetheless, this three-year period is valuable in that it offered a snapshot of changes in health and a sense of differences across individuals in the rate of health decline. Fourth, we were unable to identify the timing of many of the experiences and exposures that we investigated. Future research would benefit from survey measures that more accurately capture the timing and severity of exposure to adversity. Finally, these analyses do not account for other possible confounders, such as genetic predisposition and community support systems, and must be interpreted with this limitation in mind.

In conclusion, this investigation has offered important insights into the associations between adversity and health outcomes in a cohort of aging adults living in an economically disadvantaged rural setting in South Africa. While we did find evidence of declining health in response to adverse exposures in some cases, our results shed light on the possibility that exposure to adversity may sometimes lead to greater improvements in health within specific time frames in later adulthood. Policymakers and researchers would benefit from future work that unpacks the specific mechanisms at play that can lead to either improved or worsened health in response to lifetime adversity. Although our results may not be generalizable to other settings and populations, they contribute to the literature by providing a more nuanced and comprehensive understanding of how various adverse exposures may impact different health outcomes within a population that has historically faced significant challenges.
