# Supplementary material for: Exposure to Adversity and its Impact on Later Life Cognitive, Mental, and Physical Health
Source: Int J Public Health. 2024 Jun 19;69:1606499. doi: 10.3389/ijph.2024.1606499 (PMC11219567; doi:10.3389/ijph.2024.1606499)
Supplement: Supplementary file 1 [file DataSheet1.pdf]

Supplemental Table S1. Ordinary least squares regression predicting decline in cognitive, mental, and physical health; Interactions by gender (Agincourt, South Africa. 2014-2022).

	Model 1<br>Decline in Cognitive Health	Model 2<br>Decline in Mental Health	Model 3<br>Decline in Physical Health
Intercept	-0.50 ***<br>(0.05)	0.20 ***<br>(0.05)	-0.44 ***<br>(0.05)
Independent variables (Exposures)			
Adverse family experiences in childhood	-0.10 ***<br>(0.02)	0.01<br>(0.02)	-0.03<br>(0.02)
X Gender	-0.11 ***<br>(0.03)	-0.01<br>(0.03)	0.02<br>(0.03)
Ever had spouse, partner or child who was an addict	-0.01<br>(0.05)	-0.07<br>(0.05)	-0.02<br>(0.05)
X Gender	-0.06<br>(0.05)	0.01<br>(0.05)	0.04<br>(0.06)
Exposure to violence (not combat)	0.13 ***<br>(0.02)	-0.03<br>(0.02)	-0.12 ***<br>(0.02)
X Gender	0.04<br>(0.03)	-0.09 **<br>(0.03)	0.02<br>(0.03)
Exposure to violence (combat)	-0.18 ***<br>(0.03)	0.14 ***<br>(0.03)	0.04<br>(0.03)
X Gender	-0.27 ***<br>(0.04)	0.25 ***<br>(0.04)	0.07 +<br>(0.04)
Experience of assault	-0.04<br>(0.03)	0.27 ***<br>(0.03)	0.04<br>(0.03)
X Gender	-0.01<br>(0.05)	-0.12 **<br>(0.04)	-0.02<br>(0.05)
Exposure to natural disaster	-0.05 **<br>(0.02)	-0.13 ***<br>(0.02)	-0.03<br>(0.02)
X Gender	-0.02<br>(0.02)	0.10 ***<br>(0.02)	-0.002<br>(0.03)
N	*3,014*	*3,062*	*3,399*

Models weighted for mortality. All covariates included, as in manuscript tables.
Beta coefficients with standard errors in parentheses
Two-tailed tests, + p<.10, * p<.05, **P<.01, *** p<.001

Supplemental Table S2. Ordinary least squares regression predicting decline in cognitive, mental, and physical health; Sample of women ages 40-49 (Agincourt, South Africa. 2014-2022).

	Model 1<br>Decline in Cognitive Health	Model 2<br>Decline in Mental Health	Model 3<br>Decline in Physical Health
Intercept	-0.80 **<br>(0.30)	-0.30<br>(0.20)	-0.02<br>(0.06)
Independent variables (Exposures)			
Adverse family experiences in childhood	-0.17 ***<br>(0.04)	-0.03<br>(0.03)	-0.01<br>(0.01)
Ever had spouse, partner or child who was an addict	0.20 ***<br>(0.06)	-0.13 ***<br>(0.04)	0.06 ***<br>(0.01)
Exposure to violence (not combat)	-0.09 +<br>(0.06)	0.01<br>(0.04)	-0.03 **<br>(0.01)
Exposure to violence (combat)	0.33 ***<br>(0.07)	0.01<br>(0.05)	0.01<br>(0.01)
Experience of assault	0.09<br>(0.07)	0.10 *<br>(0.05)	-0.03 +<br>(0.01)
Exposure to natural disaster	-0.05<br>(0.04)	-0.02<br>(0.02)	0.02 **<br>(0.01)
N	*372*	*376*	*392*

Models weighted for mortality. All covariates included, as in manuscript tables, except retired now becomes part of employment status reference group.
Beta coefficients with standard errors in parentheses
Two-tailed tests, + p<.10, * p<.05, **P<.01, *** p<.001

Supplemental Table S3. Ordinary least squares regression predicting decline in cognitive, mental, and physical health; Sample of women ages 75+ (Agincourt, South Africa. 2014-2022).

	Model 1<br>Decline in Cognitive Health	Model 2<br>Decline in Mental Health	Model 3<br>Decline in Physical Health
Intercept	-1.28 **<br>(0.42)	0.06<br>(0.05)	-4.53 ***<br>(0.47)
Independent variables (Exposures)			
Adverse family experiences in childhood	-0.20 ***<br>(0.05)	0.01<br>(0.01)	0.08<br>(0.07)
Ever had spouse, partner or child who was an addict	-0.08<br>(0.08)	-0.03 **<br>(0.01)	0.16<br>(0.11)
Exposure to violence (not combat)	0.29 ***<br>(0.08)	-0.03 **<br>(0.01)	-0.23 *<br>(0.09)
Exposure to violence (combat)	-0.47 ***<br>(0.08)	0.04 ***<br>(0.01)	0.43 ***<br>(0.10)
Experience of assault	-0.11<br>(0.09)	0.02 +<br>(0.01)	0.06<br>(0.11)
Exposure to natural disaster	0.22 ***<br>(0.05)	0.03 ***<br>(0.01)	-0.11 +<br>(0.06)
N	*208*	*215*	*279*

Models weighted for mortality. All covariates included, as in manuscript tables except primary and secondary education are combined into one variable.
Beta coefficients with standard errors in parentheses
Two-tailed tests, + p<.10, * p<.05, **P<.01, *** p<.001

Supplemental Table S4. Ordinary least squares regression predicting decline in cognitive, mental, and physical health; Sample of men ages 40-49 (Agincourt, South Africa. 2014-2022).

	Model 1<br>Decline in Cognitive Health	Model 2<br>Decline in Mental Health	Model 3<br>Decline in Physical Health
Intercept	0.25<br>(0.34)	-1.13 *<br>(0.52)	-0.62 *<br>(0.30)
Independent variables (Exposures)			
Adverse family experiences in childhood	-0.38 ***<br>(0.05)	-0.11<br>(0.07)	-0.01<br>(0.04)
Ever had spouse, partner or child who was an addict	0.07<br>(0.14)	-0.22<br>(0.22)	1.30 ***<br>(0.10)
Exposure to violence (not combat)	-0.11 *<br>(0.05)	0.01<br>(0.08)	-0.11 *<br>(0.04)
Exposure to violence (combat)	0.08<br>(0.07)	-0.10<br>(0.10)	-0.07<br>(0.06)
Experience of assault	0.30 ***<br>(0.07)	0.27 *<br>(0.11)	0.36 ***<br>(0.06)
Exposure to natural disaster	-0.08 *<br>(0.04)	-0.25 ***<br>(0.06)	0.07 *<br>(0.03)
N	*240*	*244*	*294*

Models weighted for mortality. All covariates included, as in manuscript tables, except retired now becomes part of employment status reference group.
Beta coefficients with standard errors in parentheses
Two-tailed tests, + p<.10, * p<.05, **P<.01, *** p<.001

Supplemental Table S5. Ordinary least squares regression predicting decline in cognitive, mental, and physical health; Sample of men ages 75+ (Agincourt, South Africa. 2014-2022).

	Model 1<br>Decline in Cognitive Health	Model 2<br>Decline in Mental Health	Model 3<br>Decline in Physical Health
Intercept	0.02<br>(0.46)	0.08 *<br>(0.04)	0.07<br>(0.59)
Independent variables (Exposures)			
Adverse family experiences in childhood	0.20 **<br>(0.07)	-0.004<br>(0.01)	0.03<br>(0.09)
Ever had spouse, partner or child who was an addict	-0.12<br>(0.12)	-0.04 ***<br>(0.01)	-0.10<br>(0.16)
Exposure to violence (not combat)	0.16 *<br>(0.08)	0.05 ***<br>(0.01)	-0.25 **<br>(0.09)
Exposure to violence (combat)	0.26 ***<br>(0.08)	0.01<br>(0.01)	0.26 **<br>(0.10)
Experience of assault	-0.32 **<br>(0.11)	-0.04 ***<br>(0.01)	-0.08<br>(0.14)
Exposure to natural disaster	0.07<br>(0.06)	-0.01 **<br>(0.01)	-0.36 ***<br>(0.08)
N	*154*	*156*	*193*

Models weighted for mortality. All covariates included, as in manuscript tables.
Beta coefficients with standard errors in parentheses
Two-tailed tests, + p<.10, * p<.05, **P<.01, *** p<.001
